# Metal-Organic Frameworks: NU-1000 a Sensing Probe for the Detection of Water Contaminants

**DOI:** 10.1063/4.0001130

**Published:** 2025-10-27

**Authors:** Nafees Iqbal, Mario Wriedt

**Affiliations:** The University of Texas at Dallas (UTD), Dallas, Texas, USA

## Abstract

Access to clean drinking water remains a global challenge, with over 1.2 billion people consuming contaminated water daily. Among the most persistent and harmful pollutants are per- and polyfluoroalkyl substances (PFAS), a class of synthetic chemicals used in nonstick cookware, cosmetics, and water-repellent materials. Short-chain PFAS are especially difficult to detect due to their high chemical stability and low concentrations in environmental samples. To address this challenge, we investigate the synthesis and application of NU- 1000, a zirconium- based metal-organic framework (MOF), as a sensing platform for short- chain PFAS in water. NU-1000 offers several advantageous properties: a very high surface area (>2000 m^2^/g), robust chemical stability, and a unique hierarchical pore structure consisting of microporous triangular channels (∼12 Å) and mesoporous hexagonal channels (∼30 Å) that promote efficient diffusion and adsorption of small contaminants. Additionally, its highly conjugated linkers exhibit strong fluorescence that changes upon PFAS interaction—enabling fast, visual detection. Our research highlights NU-1000’s potential as a selective, regenerable, and field-deployable material for detecting PFAS, offering a practical alternative to instrumentation-heavy methods currently used in environmental monitoring.

